# Transitioning to Low-Carbon Residential Heating: The Impacts of Material-Related Emissions

**DOI:** 10.1021/acs.est.1c06362

**Published:** 2022-05-12

**Authors:** Teun Johannes Verhagen, Hale Cetinay, Ester van der Voet, Benjamin Sprecher

**Affiliations:** † Institute of Environmental Sciences (CML), Leiden University, Einsteinweg 2 (Bio-Science Park), 2333 CC Leiden, The Netherlands; ‡ Faculty of Industrial Design Engineering, TU Delft, Landbergstraat 15, 2628 CE Delft, The Netherlands

**Keywords:** heating technologies, low-carbon, material impact, cradle-to-gate emissions

## Abstract

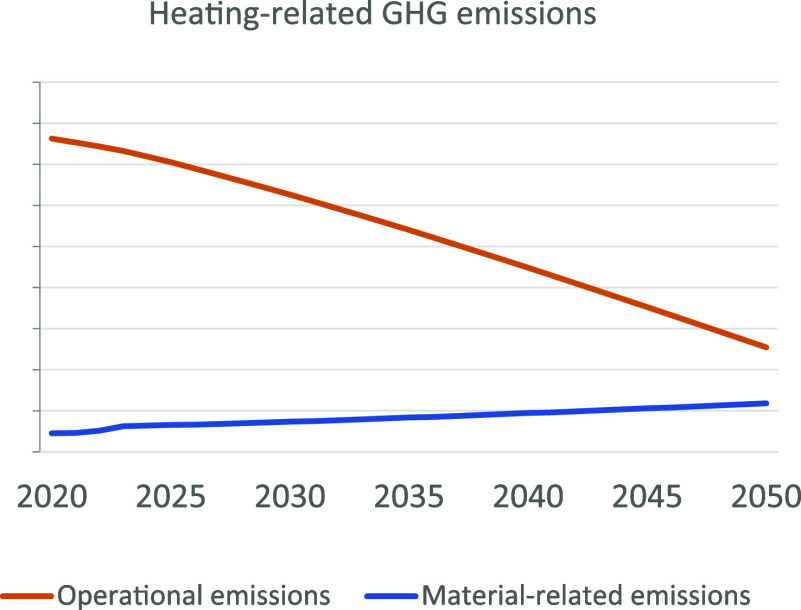

To achieve climate neutrality, future urban heating systems will need to use a variety of low-carbon heating technologies. The transition toward low-carbon heating technologies necessitates a complete restructuring of the heating system, with significant associated material requirements. However, little research has been done into the quantity and environmental impact of the required materials for this system change. We analyzed the material demand and the environmental impact of the transition toward low-carbon heating in the Netherlands across three scenarios based on the local availability and capacity for sources of low-carbon heat. A wide range of materials are included, covering aggregates, construction materials, metals, plastics, and critical materials. We find that while the Dutch policy goal of reducing GHG emissions by 90% before 2050 can be achieved if only direct emissions from the heating system are considered, this is no longer the case when the cradle-to-gate emissions from the additional materials, especially insulation materials, are taken into account. The implementation of these technologies will require 59–63 megatons of materials in the period of 2021–2050, leading to a maximum reduction of 62%.

## Abstract 2

These findings are relevant to understanding the environmental impact of a country-wide transition toward low-carbon residential heating technologies.

## 1 Introduction

The worldwide heating demand for buildings and associated greenhouse gas (GHG) emissions has in recent years been increasing at a rapid pace, currently accounting for 6% of global GHG emissions. The annual global heat consumption of buildings has increased from 26 EJ in 2000 to 32 EJ in 2020.^1,2^ In line with the Paris agreement, the Dutch government set the goal to reduce its national heating-related GHG emissions by 90% before 2050 (compared with 1990).^3^ The Dutch heating system is predominantly natural gas-based, unlike in many other countries, making it an interesting contemporary case study.^4^ To achieve the goal of the Dutch government, it is crucial to transition the Dutch natural gas-based heating system to low-carbon heating technologies, which we will refer to as the heating transition (“*warmtetransitie*” in Dutch).

The heating transition is one element of the larger energy transition, which has been studied extensively from the perspective of material intensity and the materials required for building up the renewable energy system and associated infrastructure.^5,6^ For example, the material stock of the electricity system will increase significantly with the development toward a renewable energy system.^5,7^ The implementation of low-carbon electricity technologies also increases the demand for metals, which have a considerable environmental impact.^8^ The energy transition will decrease the operational GHG emissions of energy generation but at the cost of an increased material intensity.^9^ This could also be true for the heating transition, but this has not been researched yet.

While previous studies have shown that operational heating-related GHG emissions can be considerably reduced by the use of low-carbon heating technologies, such as heat pumps and heating networks,^10,11^ the transition to a low-carbon heating system also has consequences for the existing heating system and its material composition. The current Dutch heating system operates on natural gas, utilizing a country-wide gas transmission network. This existing heating system, including in-house heating, infrastructure, and energy production, will have to be adapted to accommodate low-carbon heating technologies that operate on different sources of heat. Low-carbon heating technologies such as low-temperature (LT) and high-temperature (HT) heating networks utilize a network of underground pipes for heat transmission, while heat pump technologies are dependent on the electricity grid and will require additional grid capacity.^12^ The implementation of these low-carbon heating technologies requires additional and different materials compared to the current heating system.^8,12−16^

It has been well-established that part of the operational GHG emission reductions achieved by low-carbon heating technologies could be undone by the increased emissions related to the production of the materials required for these technologies.^8,17−20^ However, to the best or our knowledge, no research has been done on the system-wide influence of the material-related emissions of this heating transition. In particular, no research has been done to assess the combined material demand of the production of low-carbon heat, the material demand of the necessary adjustments to residential buildings, and the material demand related to the energy infrastructure required for the implementation of low-carbon heating technologies.

This work assesses the material-related cradle-to-gate emissions of the future Dutch heating system and integrates this assessment with the outcomes of previous work on the operational emissions of the future Dutch heating system. We analyze the feasibility of the Dutch policy goal with three future development pathways of the Dutch heating system, based on the local availability and capacity for sources of low-carbon heat.

## 2 Methods and Data

Four low-carbon heating technologies commonly found in the literature and policy documents were selected. We searched gray and scientific literature for information on the quantities of materials required for the implementation of these four low-carbon heating technologies in residential buildings in the Netherlands, supporting infrastructure, and the corresponding production of electricity or heat. The BAG, a Dutch governmental dataset with information on building types and floor areas, was used to determine the number of residential buildings and dwellings in the Netherlands.^21^ Subsequently, this information was then used to calculate the materials required for each low-carbon heating technology per residential building.

**Figure 1:**
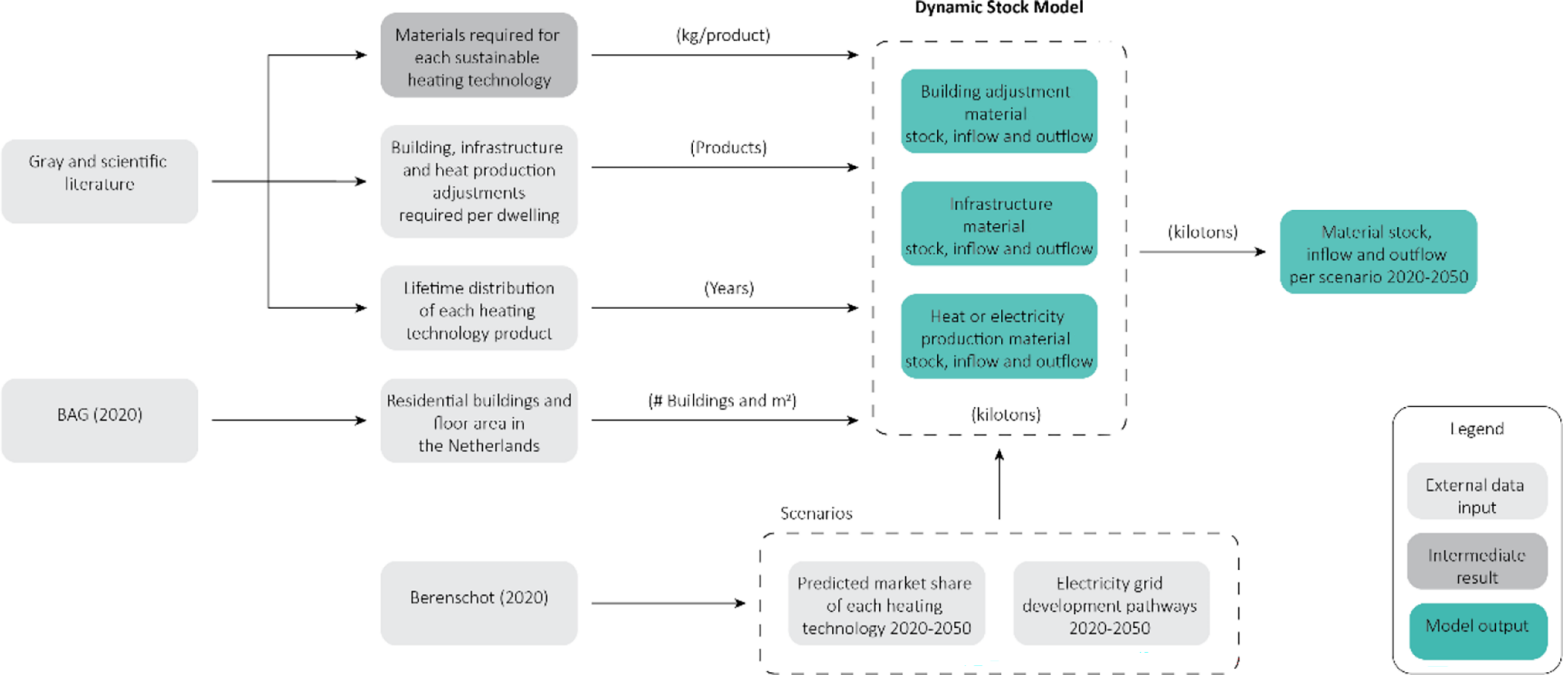
Conceptual outline of the model.

Three scenarios of the material stock and inflow for the future Dutch heating system were modeled with a dynamic stock model. They were based on the scenarios by Berenschot.^22^ For the in- and outflow of materials, we used lifetime distributions from the literature for each subcomponent of low-carbon heating technologies (see Section 2.5 and Table S3 in the SI for the detailed overview). The three scenarios also included assumptions on the composition of the future electricity grid, which we included in our model. Next, the cradle-to-gate emissions in CO_2_-equivalent (CO_2_-eq) of the materials were calculated for each scenario. Lastly, these emissions were integrated with the operational emissions in CO_2_-eq of the heating system, which we assessed in a previous publication,^11^ to arrive at an estimate for total emissions in CO_2_-eq related to Dutch residential heating. In addition, we compared the system-wide emission impact of this transition toward low-carbon heating with the operational emissions of the current natural gas-based system. The conceptual outline of the model is given in Figure 1. The material intensity values for the implementation of each heating technology can be found in Supporting Information III.

### 2.1 Low-Carbon Heating Technologies and Model Assumptions

The following technologies were analyzed in this research: high-temperature (HT) heating networks, low-temperature (LT) heating networks, heat pumps, and hybrid heat pumps. We based this selection on our earlier study and added hybrid heat pumps.^11^ We analyzed three subcomponents of the Dutch heating system: the required building adjustments (for example, the heat pump, insulation, floor heating, and low-temperature radiators), the infrastructure required (heating network, electricity grid, etc.), and the heat and electricity production (geothermal district heating, solar panels, windmills, and biogas-fired power plant). For all technologies except the HT heating networks, we also assumed the installation of floor heating in the buildings. We segregated the residential buildings in the Netherlands into five building types: apartments, corner houses, terraced houses, semidetached houses, and detached houses. This corresponds to the classification used in the BAG-GIS dataset, which was also used to calculate the number of dwellings and the floor area per building type.

For LT and HT heating networks, we based the in-house and infrastructure adjustments on the study by Oliver-Solà et al.^19^ We assumed the installation of a heat exchanger, in-house distribution pipes for the heating network heat, and the installation of an HT or LT heat network on a neighborhood scale.^23,24^ Based on the report by Berenschot, the heat production of HT heating networks was assumed to be waste heat from a biogas-fired power plant, while for LT heating networks, we assumed the use of geothermal heat.^25^ In the Netherlands, geothermal heat is extracted from wells reaching a depth of 2,000 meters.^26^ For the production of HT waste heat, we assumed that gas-fired power plants operate on biogas or hydrogen in the future and that these power plants have a consistent material composition.^27−29^

The most widely implemented heat pump technology in the Netherlands is the air-to-water heat pump since its initial investment is lower in comparison with water-to-water heat pumps.^30^ We used the study by Greening and Azapagic for our material inventory of a 10 kW air-to-water heat pump.^17^ For every building type except apartments, we assumed the installation of a heat pump for each separate dwelling. For apartments, we assumed a heat pump for every 150 m^2^ of floor area, as a heat pump can be shared across multiple dwellings in an apartment building. The use of air-to-water heat pumps influences the composition of the electricity grid,^31,32^ which we further discuss in Section 2.2. For the electricity used by the heat pumps, we assumed a combination of biogas-fired power plants, onshore and offshore windmills, and PV panels as specified by Berenschot.^33^

For the material inventory of hybrid heat pumps, we also used the study by Greening and Azapagic. We scaled down the material inventory from the 10 kW heat pump used in the source to a heat pump with a smaller 6 kW capacity used in the hybrid heat pump technology.^17^ Furthermore, we also included the material inventory of a small CV boiler from the study by Oliver-Solà et al.^34^ Just as our air-to-water heat pump assumptions, the use of a hybrid heat pump influences this grid composition as discussed in Section 2.2. We also modeled an identical composition of electricity production technologies and the shared use of hybrid heat pumps in apartment buildings. For the peak boiler in the hybrid heat pumps, we assumed the use of natural gas as the energy source as it is still unclear whether there will be enough future production capacity in the Netherlands of renewable gases such as hydrogen or biogas.^22^

### 2.2 Material Demand for the Implementation of Low-Carbon Heating Technologies

The material demand for the implementation of low-carbon heating technologies was calculated in kg of material required per dwelling connected to the Dutch heating system (shown in Section 3.1 of the Results section). This included the building adjustments, infrastructure extensions, and the additional required heat or electricity production. An overview of the materials included in the model is given in Table S5 of Supporting Information II.

The sources of the required materials for the implementation of low-carbon heating technologies ranged from scientific literature to gray literature and the Ecoinvent database. We used the Ecoinvent database and the literature to assess the materials included in, for example, a heat pump or geothermal district heating. For each technology and subcomponent, we calculated their cradle-to-gate emissions in CO_2_-eq based on their material composition. The packaging materials were excluded from our material inventory. An overview of the materials included and quantified in our model is given in Supporting Information II, and the detailed specification of materials required per heating technology connection per household is provided in Supporting Information III.

For the inclusion of insulation suitable for low-carbon heating technologies in Dutch buildings, we used the study by Koezjakov et al.^20^ We included expanded and extruded polystyrene (ground floor and foundation), mineral wool (roof and walls), polyurethane foam (ground floor and façade), and wood fiberboard (foundation and façade). For the heating network technologies, we included the foundation, façade, roof, and wall insulation options. For the (hybrid) heat pump technologies, we included all the mentioned insulation materials as heat pumps require a higher degree of insulation to operate efficiently. These materials were also calculated in kg of material required per dwelling connected to the Dutch heating system for each technology.

### 2.3 Consequences of the Heating Transition for the Electricity Demand

This transition toward low-carbon heating technologies in the Netherlands resulted in an increased consumption of electricity. The existing electricity grid will have to be reinforced to accommodate the additional load of heat pumps and hybrid heat pumps.

To calculate the impact of the heat pump and hybrid heat pump integration into the electrical grid, we modeled a case study in a typical European low-voltage (lv) grid. First, we calculated the current demand in the grid by modeling the average electricity consumption of households within a theoretical city district. This model provides the basis for the operating conditions of the electrical grid before the electrification of heating systems. Next, we started integrating heat pumps into the grid and analyzed the changes in the demand and operating conditions of the grid. We calculated the materials for the addition of the heat pump technologies based on the required increase in lv grid capacity. In Supporting Information I, we discuss the steps of this analysis of the electricity demand development and the results in detail.

### 2.4 Development Pathways of the Dutch Heating System

We included three scenarios on the composition of the future Dutch heating system in our analysis: a mixed scenario with mainly LT heating networks and heat pumps, a high heat pump scenario, and a high hybrid heat pump scenario. Our scenarios were based on the warmtescenario report by Berenschot, which explores multiple heating system pathways for the Netherlands from 2020 to 2050.^22^ In their analysis, the local availability and capacity for sources of low-carbon heat were considered. Even though each scenario has a different dominant technology, their market share composition does not differ that much (overview in Table S4a in Supporting Information II). For each of our three scenarios, we varied the electricity generation composition to simulate different developments based on the klimaatneutrale energiescenarios or climate-neutral energy scenarios report by Berenschot (overview in Table S4b in Supporting Information II).^33^ In the absence of time series information, we assumed a linear increase in low-carbon heating technologies’ market share over time to replace the existing natural gas heating system. The scenarios only explored the composition of the future Dutch heating system. In the Berenschot report, no variation in the total heating demand between different scenarios was assumed. Although having variations in heating demand might have made the scenarios more differentiated, we chose to not adapt the scenarios for our work, as that would negatively influence the ability of policy makers to consider our work together with the outcomes of the Berenschot report.

### 2.5 Dynamic Stock Modeling

In this section, we describe how we estimated the stocks and in- and outflow of materials for each heating technology. They were based on the number of dwellings using a low-carbon heating technology, the materials required for the implementation of the low-carbon technologies per dwelling, and their expected lifetime. The Dutch population growth expectation from 2021 to 2050 was used to estimate the increase in dwellings over time.^35^ With a stock-driven dynamic stock model, we calculated the material demand (inflow), outflow (waste), and in-use stock over time related to the Dutch heating system, from 2020 to 2050, for each heating technology subcomponent (building adjustments, infrastructure, and heat or electricity production).^36^ Based on the stock, we determined the in- and outflow with a distributed life span (*L*) using a Weibull function. In the model, for the calculation of a stock (*S*) and in- and outflow at certain years (*t*), the following function was used:
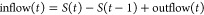
1

For the calculation of the material stock over time, we multiplied the number of dwellings utilizing low-carbon heating technologies with the materials required for each heating technology subcomponent (building adjustments, infrastructure, and energy production). The sum of these three subcomponents is the total amount of materials required per dwelling for a low-carbon heating technology. The subcomponents of a system get only replaced based on their own lifetimes. The in- and outflow of materials for each year were calculated based on the subcomponents and their average lifetime distribution with the dynamic stock model, resulting in the following formula:
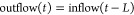
2

The inflow was calculated as the difference between the addition to the stock and the calculated outflow in a year. When Weibull distribution parameters were not available, we used standard Weibull distribution values based on the average lifetime distributions of the subcomponents of the heating system. Stock accumulation models are mainly sensitive to the average lifespan and almost insensitive to the choice of lifespan distribution function.^37^ In Table S3 of Supporting Information II, an overview of the mean lifetimes and sources for each low-carbon heating technology subcomponent is given.

### 2.6 Operational and Cradle-to-Gate Emissions of Low-Carbon Heating Technologies

For the system-wide analysis of the Dutch future heating system, we quantified the operational and the cradle-to-gate GHG emissions measured in kg CO_2_-eq of the material inflow over time from 2021 to 2050. To calculate the cradle-to-gate impact of the materials, we used the EcoInvent 3.4 database and CMLCA 6.1 software. We only looked at the impact of the production of the materials present in a product but not at the production of the product itself.

The operational greenhouse gas (GHG) emissions were based on Verhagen et al.’s study and reported in kg of annual CO_2_-eq per heating technology. This impact includes the emissions from electricity that replace natural gas-based emissions. We used the average heat consumption in kWh per dwelling in the Netherlands in 2020. We also included improvements from insulation, lowering the average energy demand for space heating in a dwelling by 60%. The total emissions produced by a low-carbon heating technology for the production of a kWh of heat were calculated in a CO_2_ intensity value. The CO_2_ intensity value included transportation losses from infrastructure and the production of heat from the corresponding sources as described in Section 2.2. As a result, the annual operational CO_2_ emissions per dwelling were calculated as follows:
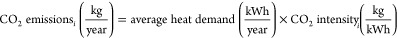
3

Based on the market share and the number of dwellings for each low-carbon heating technology (*i*), the total operational emissions per scenario were calculated. In Section 3.5, we assessed the cradle-to-gate and operational emissions from 2021 to 2050. We also included the operational emissions in CO_2_-eq of natural gas-based heating systems based on the paper by Oliver-Solà et al. as a business-as-usual (BAU) scenario.^34^ For this scenario, we assumed that 95% of the Dutch households keep utilizing the natural gas-based heating system.

## 3 Results

### 3.1 Material Requirements and Cradle-to-Gate Emissions of Low-Carbon Heating Technologies per Dwelling

**Figure 2:**
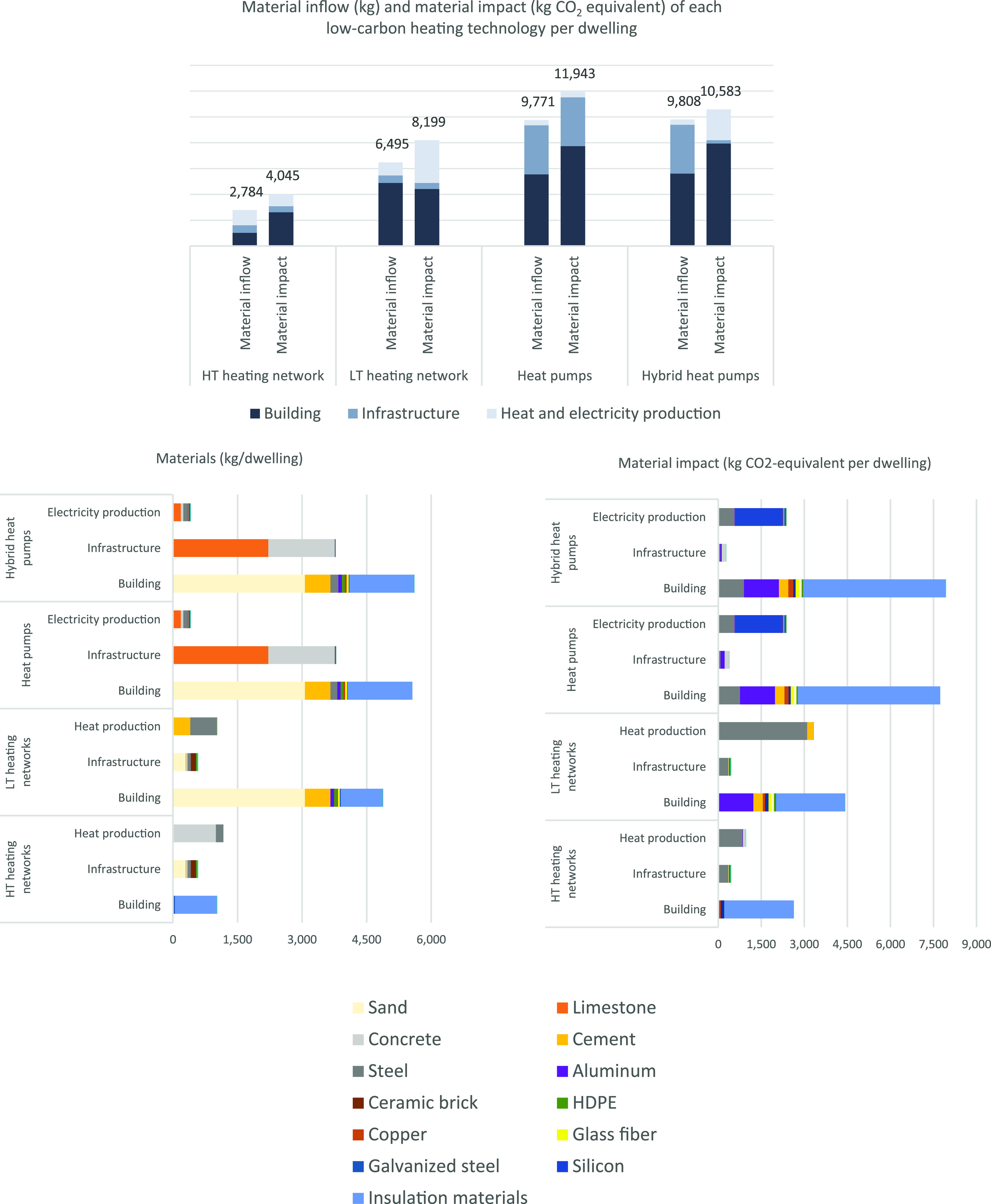
Low-carbon heating technology material demand (left) and cradle-to-gate emissions (right) per dwelling.

The most material-intensive technologies are the heat pump and hybrid heat pump technologies. For these two technologies, the majority of the material requirements is due to the infrastructure and building adjustments, while a small fraction of their material requirements results from heat and energy production. For the LT and HT heating network technologies, the largest share of their material requirements is generated by building adjustments and the required heat production. The material requirements of HT heating networks are substantially lower than the material demand of the other technologies due to the absence of floor heating. Overall, the aggregated material requirements for implementing low-carbon heating technologies per dwelling vary from 2,784 kg for HT heating networks to 9,808 kg for hybrid heat pumps (Figure 2).

For the cradle-to-gate impact of the material requirements, we found 2,784 kg CO_2_-eq for the HT heating technologies, 8,199 kg CO_2_-eq for the LT heating networks, 10,583 kg CO_2_-eq for the hybrid heat pumps, and 11,943 kg CO_2_-eq for the heat pumps. For all the low-carbon heating technologies, the required installation results in a significant share of their cradle-to-gate impact.

The materials with the highest emission impact are steel, insulation materials, aluminum, and silicon. The heat production for LT heating networks has a relatively high cradle-to-gate impact due to the amount of steel used in geothermal district heating. Furthermore, the infrastructure category has the lowest material impact of all technologies. The highest emissions are not always generated by the materials with the highest inflow. The largest share of the material requirements is generated by limestone, sand, and concrete, while the highest share of the emissions is caused by steel, insulation materials, aluminum, and silicon. For example, LT heating network and hybrid heat pump technologies have a comparable material impact (8,199 up to 10,583 kg CO_2_-eq) per dwelling, while the LT heating network technology has a 40% lower total material requirement in comparison with the heat pump technology (6,495 vs 9,808 kg).

### 3.2 Material Stock and Composition of the Dutch Heating System in 2050

**Figure 3:**
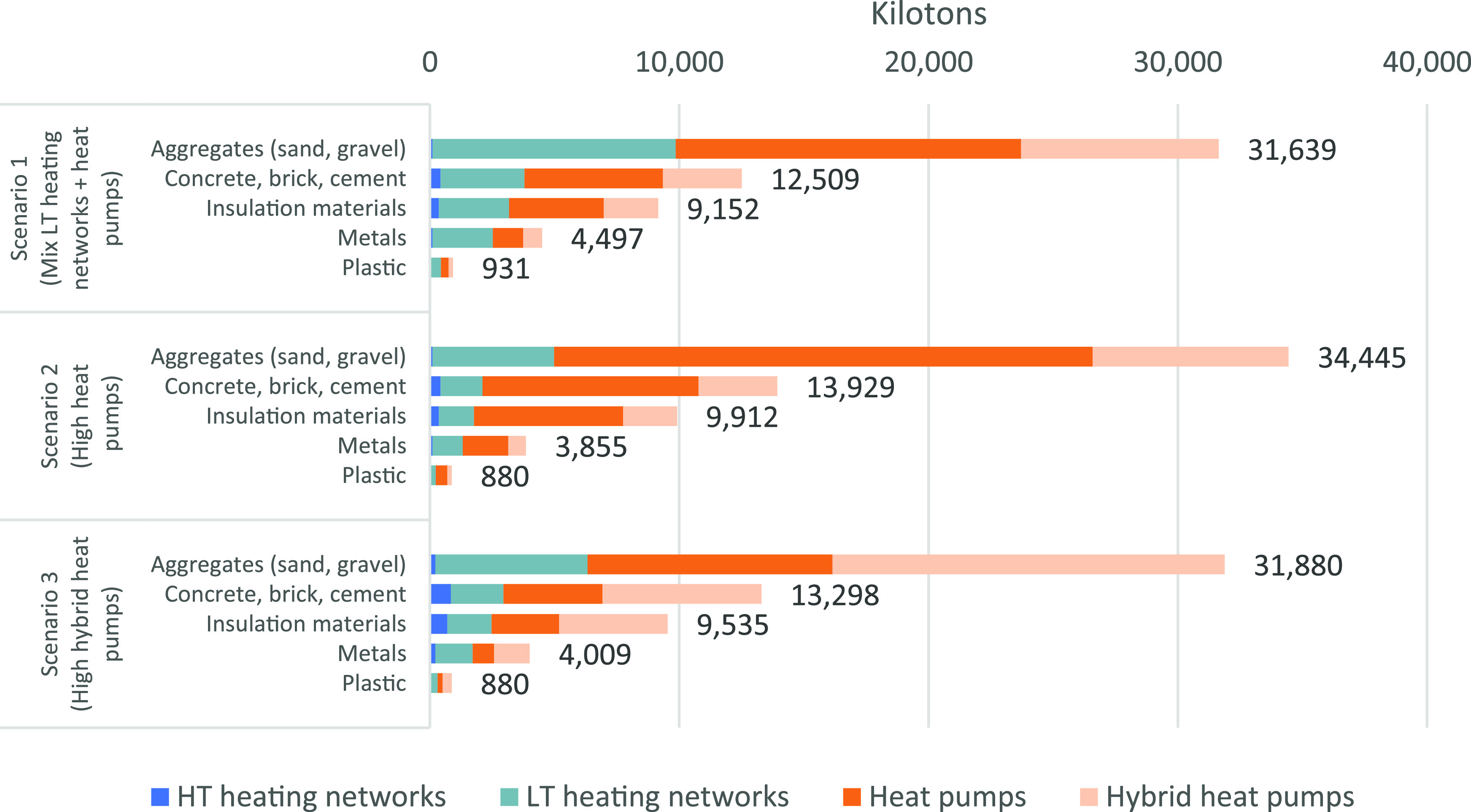
Material stock of the Dutch heating system in 2050 for each scenario and material category.

In 2050, the material stock of the Dutch heating system varies per scenario from 58,727 kilotons for scenario 1 (mixed LT + HP) and 59,603 kilotons in scenario 3 (high HHP) to 63,020 kilotons for scenario 2 (high HP). The largest material category with 31,639 up to 34,445 kilotons in the Dutch heating system composition in 2050 is aggregates (sand and gravel). The second largest category of materials with 12,509 up to 13,929 kilotons is concrete, brick, and cement. The insulation materials range from 9,152 to 9,912 kilotons per scenario. Metals and plastics are a smaller material category in the future Dutch heating system with 3,855–4,497 kilotons for metals and 880–931 kilotons for plastics. Overall, between scenarios, there is only a slight variation in the material stock (Figure 3).

A large share of the material stock in each scenario originates from the heat pumps and hybrid heat pumps. Most of the material stock comes from sand, insulation materials, concrete, and cement in the in-house floor heating, insulation requirements, and the transformer buildings for heat pumps and hybrid heat pumps. The largest material demand in scenarios 1 and 2 and the highest share of metal demand in every scenario originate from the LT heating networks. This results from the steel intensity of the geothermal heat source for the LT heating networks. We included all the material stock values per technology and scenario in Supporting Information III.

### 3.3 Material Inflows Related to the Dutch Heating Transition from 2020 to 2050

**Figure 4:**
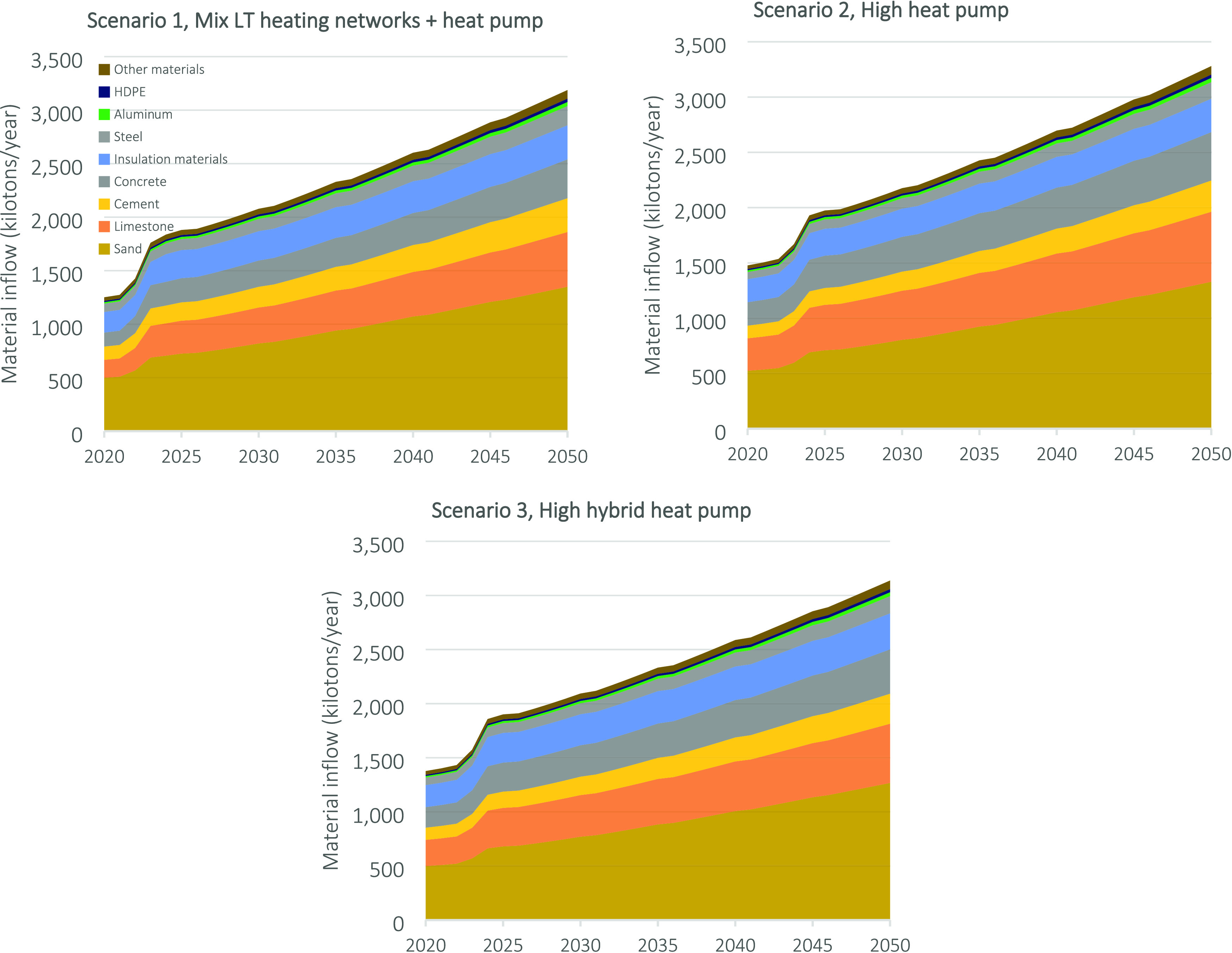
Annual material inflow and inflow composition of the Dutch heating system until 2050.

Overall, the share of materials in the inflow only differs slightly between each scenario, as illustrated by Figure 4. The annual material inflow of the low-carbon Dutch heating system is expected to increase from 1,248 to 1,476 kilotons in 2020 up to 3,137 to 3,285 kilotons in 2050 across the three heating technology scenarios.

The major material inflows result from sand, limestone, cement, concrete, insulation materials, steel, aluminum, and HDPE. Smaller inflows consist of copper, glass fiber, etc. with an inflow of around 30 up to 80 kilotons per year. The annual material inflow is generally comparable in weight and composition for each scenario. The higher relative share of steel and aluminum in scenario 1 is due to the higher share of LT heating networks in this scenario. The other inflows will largely remain the same across the scenarios. More detailed information on all material inflows can be found in Supporting Information III.

### 3.4 Cumulative Cradle-to-Gate GHG Emissions of Low-Carbon Heating Technologies from 2020 to 2050

Due to the high material inflow of scenario 3, this scenario is the most GHG-intensive option with 70.8 megatons of cradle-to-gate emissions in CO_2_-eq. The GHG emissions of the other scenarios range from 59.7 megatons of CO_2_-eq for scenario 2 up to 67.0 megatons CO_2_-eq for scenario 1. The highest cradle-to-gate impact is generated by building adjustments and electricity and heat production, while the infrastructure adjustments have the lowest impact.

**Figure 5:**
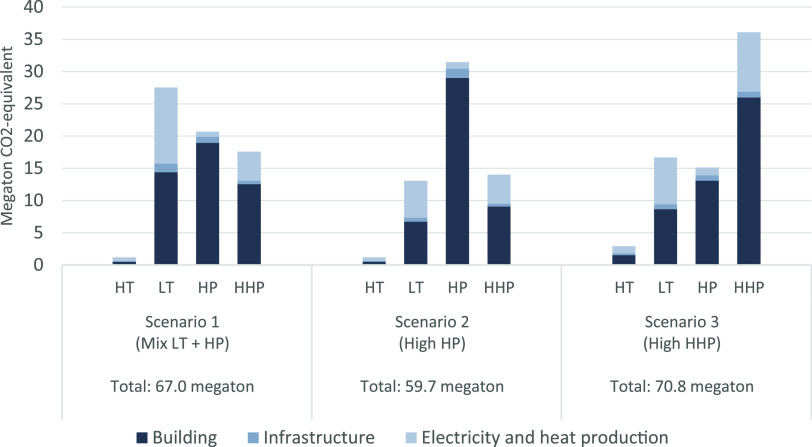
Cumulative cradle-to-gate emissions of low-carbon heating technologies’ material inflow for each scenario in the period of 2020–2050.

A trend can be observed among the heat pump and heating network technologies: most of the heat pump technologies’ impact is generated by the building adjustment category resulting from the installation of a (hybrid) heat pump and insulation requirements, while the heating network technologies’ impact is largely determined by its heat production (Figure 5). Also, the steel intensity of the LT heating network technology heat production is reflected in its emissions, as steel has a relatively high cradle-to-gate impact.

### 3.5 System-Wide GHG Emission Reduction of the Dutch Heating System

**Figure 6:**
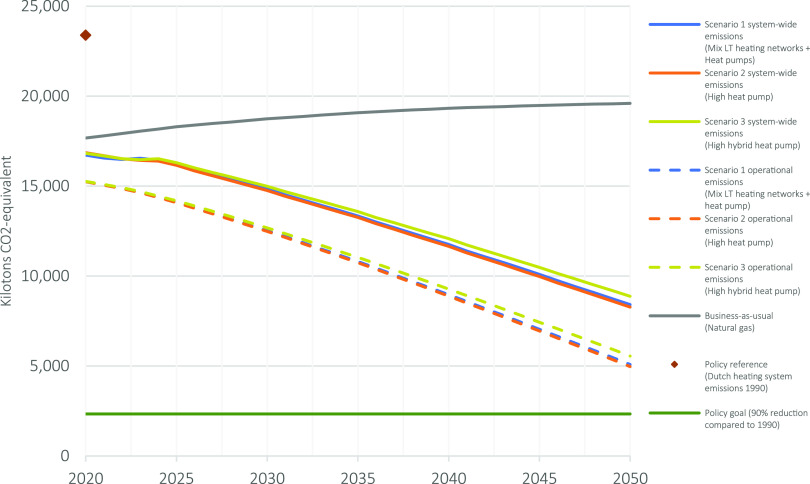
Annual Dutch heating system GHG emissions for each scenario in the period of 2020–2050.

Figure 6 shows that the highest annual net CO_2_-eq impact reduction with 15,115 kilotons in 2050 can be achieved with scenario 2 (high heat pump). In comparison with the operational emissions of the Dutch heating system in 1990, this translates to a reduction of 64%. Scenario 1 has the second highest annual emission reduction in 2050 with 14,976 kilotons, and scenario 3 has the lowest annual net CO_2_-eq impact reduction with 14,514 kilotons. In comparison with the operational emissions of the Dutch heating system in 1990, this translates to a 62–64% reduction. Furthermore, in 2050, the total cradle-to-gate impact of the in-use material stock is between 3,223 and 3,329 kilotons and will generate around 40% of the GHG emissions of the Dutch heating system. After 2050, the buildup of the Dutch low-carbon heating system is assumed to be complete. The material inflow and the corresponding cradle-to-gate impact will be reduced and largely consist of stock maintenance.

Overall, the market share of heating technologies and electricity grid development does not differ much between scenarios. Consequently, the system-wide emissions change little between the scenarios. The system-wide CO_2_ emissions are still largely determined by the operational emissions, even in 2050. Still, the share of material-related emissions will increase over time in comparison with the operational emissions.

## 4 Discussion

This work quantifies the material demand and stock as well as the cradle-to-gate CO_2_ emissions resulting from the implementation of low-carbon heating technologies in the Netherlands. We compare this to the Dutch climate goal of reducing CO_2_ emissions by 90% before 2050 from the 1990 baseline. We used three future scenarios based on the local availability and capacity of low-carbon heat. This research is a continuation of earlier work, which assessed the operational emissions of the future Dutch heating system, allowing for a comparison of both the operational and cradle-to-gate emissions.^11^

Taking into account emissions related to materials has major consequences for the achievability of the Dutch climate goals. Across all three scenarios of a future Dutch heating system, an operational emissions-only point of view would lead to the conclusion that an 80% reduction will be achieved. However, the additional material requirements negate part of the emission reduction benefits of the heating transition, to the point that a reduction in system-wide GHG emissions of not more than 62–64% is achievable.

The stated policy goal of reducing urban heating-related GHG emissions by 90% in 2050 is achievable but only with the right combination of heating technologies and sources of heat.^11^ Furthermore, the heating system will have to be designed with technologies that have a significantly lower material demand. In comparison with the existing heating system, some parts of the low-carbon heating system are less material-intensive. For example, older residential buildings in the Netherlands use heavy iron piping systems, radiators, and boilers for the distribution of heat. More modern solutions allow the utilization of lightweight polymer-based distribution systems, smaller radiators, and smaller boilers. Insulation materials with lower overall life-cycle impacts must be developed. Innovation and dematerialization of heating systems could alleviate some of the material demand and the corresponding environmental impact of this transition toward low-carbon heating.

The material inflows and associated cradle-to-gate GHG emissions of the Dutch low-carbon heating system will decrease after 2050, as the stock will transition from a growth state to a maintenance state. Still, we find that the share of material-related emissions will increase to 40% of the heating system-wide emissions in 2050. These results are similar to those of Xining and Steubing and Koezjakov et al., who found out that the embodied emissions of building materials will increase from 10–12% in the current situation to 36–46% of the total lifetime emissions in energy-efficient homes.^4,20^

Contrarily to expectations, this study did not find a significant difference in the material demand between the different scenarios planned by the Dutch government due to the comparable material demand of the low-carbon heating technologies. Therefore, the choice of a combination of low-carbon heating technologies will have to be based on other considerations, such as the availability of sources of heat.

The overall amount of materials invested in the heating system from 2020 to 2050 is 60–70 megatons. The materials with the highest share of the cradle-to-gate CO_2_ emissions are insulation materials, steel, aluminum, and silicon, while in terms of weight, sand, limestone, and concrete constitute a large share of the annual material demand. Critical materials included in our research amounted to less than 0.1% of the material mass and less than 1.5% of the cradle-to-gate impact. Floor heating for low-temperature space heating requires a considerable amount of sand and concrete, while geothermal heat plants are steel-intensive methods for heat production.

The Dutch heating system is estimated to have a stock of around 3.3 up to 4.2 million tons of steel in 2050 and an annual metal inflow of 180 up to 250 kilotons per year. This means that the Dutch heating system is 4–5 times less metal-intensive than the future Dutch electricity system, which will comprise a material stock of around 14,300–25,800 kilotons of steel in 2050 and an annual metal inflow of 800 up to 1,600 kilotons per year.^38^ Furthermore, we found an annual inflow of concrete in the Dutch low-carbon heating system of around 254 up to 679 kilotons per year or an order of magnitude less than the concrete inflow of the Dutch building sector, which according to a study by Zhang et al. amounts to around 2,800 up to 4,800 kilotons per year.^6^

An uncertainty exists over the future composition of the Dutch low-carbon heating system. Several scenarios were used to address this uncertainty. While the differences in the market share between the different low-carbon heating technologies in these scenarios are limited, the material flows will remain largely the same even with a different composition of the market share of these technologies. This is due to the comparable material demand of the low-carbon heating technologies. There is also an uncertainty over the average lifetime distributions for the subcomponents of the future Dutch heating system. The choice of lifetime distribution function has little influence on stock accumulation models.^37^ On the other hand, the size of the material inflow and the generation of waste streams are sensitive to the average lifespan of these subcomponents. Therefore, the use of different lifetime distributions for the subcomponents of the heating system will influence the size of the material inflow and the cradle-to-gate impact.

The modeling of the electricity system carries more uncertainties. In addition to the transition toward low-carbon heating technologies, the future electricity grid capacity will also be influenced by other developments such as the energy transition, increased cooling demand, and further adoption of electric cars.^39^ We modeled an increase in low-voltage and medium-voltage grid capacity based on the additional grid load and the corresponding materials necessary for the implementation of heat pumps. It is possible that we overallocated the share of this material demand for the transition toward low-carbon heating in our research due to potential overlap of the additional grid capacity with the other developments. Furthermore, heat pumps can also provide cooling. With an increasing demand for residential cooling in the Netherlands, the utilization of heat pumps could prevent or replace independent cooling solutions and the corresponding materials.

A limitation of this research is the use of a cradle-to-gate impact assessment rather than a full life-cycle assessment. Due to the broad variety of materials used in the model, it was not possible to include the full life cycle of all the included materials. Furthermore, there is a limited number of available sources on the material data of the low-carbon heating technologies used in this research. With more data on materials in low-carbon heating systems, it would have been easier to model the material demand scenarios more accurately.

In this paper, we have explored the material requirements for a new, renewable-based heating system. Another related topic would be to investigate the old, fossil fuel-based heating system, which will become obsolete over time. We could explore possible end-of-life pathways, to see in what way we could make the best use of this urban mine.

## Supporting Information Available

Consequences of the heating transition for the electricity demand in detail (I) and input data used in the model (II) (PDF)
Output data of the model per scenario and materials per technology (III) (ZIP)
