# Supplementary material for: Transitioning to Low-Carbon Residential Heating: The Impacts of Material-Related Emissions
Source: Environ Sci Technol. 2022 May 12;56(12):8561–70. doi: 10.1021/acs.est.1c06362 (PMC9228087; doi:10.1021/acs.est.1c06362)
Supplement: Supplementary file 1 — es1c06362_si_001.pdf [file es1c06362_si_001.pdf]

Transitioning to low-carbon residential heating: the impacts of material related emissions - Supplementary Information

Teun Johannes Verhagen*[(a)], Hale Iyicil Cetinay [(a)], Ester van der Voet [(a)], Benjamin Sprecher [(b)]

a. Leiden University, Institute of Environmental Sciences (CML), Einsteinweg 2 (Bio-Science park), 2333 CC Leiden, The Netherlands

b. TU Delft, Faculty of Industrial Design Engineering, Landbergstraat 15, 2628 CE Delft, The Netherlands

***Corresponding author:** E-mail: t.j.verhagen@cml.leidenuniv.nl

Pages: 8

Figures: 2

Tables: 6

In this document, we included the consequences of the heating transition for the electricity demand in detail (I), the input data used in the model (II), excluded materials (III), and the output data of the model per scenario (IV).

Supplementary Information I - Consequences of the heating transition for the electricity demand

Figure represents a typical low voltage grid in a neighbourhood in Europe [1]. The network has $N = 906$ lv connections (nodes), that are connected by $L = 905$ lv cables (links) and 1 mv/lv transformer. Figure is the graphical representation of the grid, where the nodes represent the lv connections and the links are the cables between the connections.

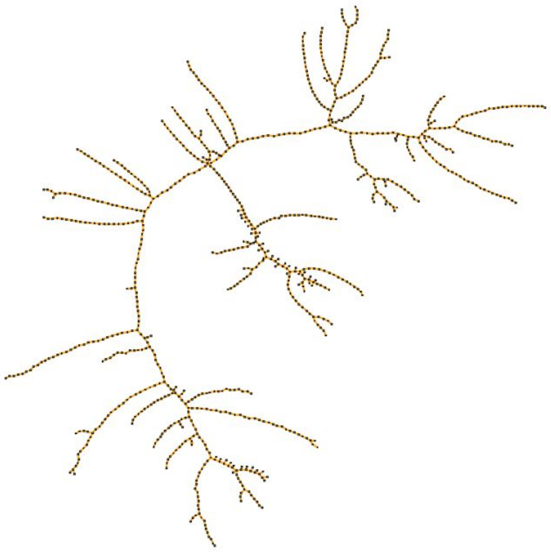

Figure S1. European low voltage grid with $N = 906$ *nodes and* $L = 905$ *links. The grid is a tree network, in other words, there are no loops. The network starts from the low voltage side of the distribution transformer (node 1).*

We use electricity consumption behavior profiles from the vereniging Nederlandse Energie-Data Uitwisseling (NEDU), which is as an umbrella organization of the Dutch electricity companies, to represent an average lv connection in the Netherlands [2]. We use the profile E1A profile, which represent a lv connection smaller than 3x25A. A typical household electricity consumption in the Netherlands is taken as 6000 kwh. The planning for the electricity grid investments are mainly based on the peak-load conditions, in other words when the electricity consumption demand is maximum. According to the NEDU profile, the peak load happens at the first week of January with the maximum peak load of 1.55 kw per household as shown in Figure .

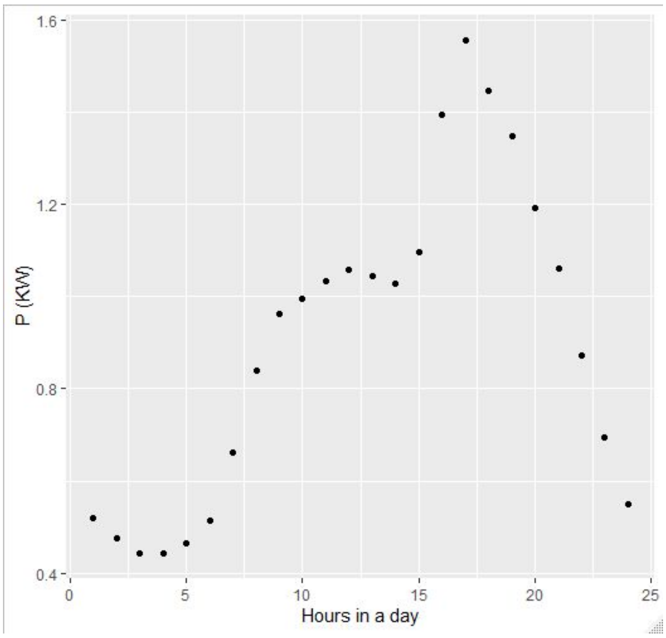

Figure S2. The electricity consumption of an average household at the peak day. The x axis represent the hours in a day whereas the y axis in the electricity demand in kw.

We will use the linearized DC power flow equations to find the flow of each link in network at the peak load conditions [3]. Following [4]. we assume that the thermal capacity c_l of a link l is $c_l = \min\{\text{mean(flows)}, (1 + \alpha) \times f_l\}$ and an investment decision is made when the flow f_l of a link is higher than its thermal capacity c_l. In this paper we take $\alpha = 0.5$.

We assume that in our test grid, initially, there are no houses with heat pumps, i.e. the number of total heat pumps is zero. Next, we add the heat pump load on the regular house hold electricity demand. We choose a heat pump of size 4 kw. Focusing at the peak day, we assume that the heat pump will work on a full capacity with COP=1 making the electrical load of a heat pump 4 kw [5]. We take the simultaneity factory of the heat pumps as 1, meaning that most people are likely have heat pump working at this winter peak day. If we the probability that a house hold getting a heat pump is h_i, the total load of a household with heat pump P_{max}^{HP} becomes

$$P_{i,max}^{HP} = P_{i,\,max}^{0} + h_i \times s_i$$

where s_i is the size of the heat pump at connection (node) i and $P_{max}^{0} = 1.55$ is the initial peak load of the connection i.

We assume a uniform distribution of the heat pump among the houses, in other words, every house has an equal probability h_i to obtain a heat pump. Under these assumptions, we re-solve the dc power flow again to calculate the flow of each link in the network and we compare

these new flows of the links with their thermal capacities to find the overloaded links. In addition, using the lengths of the cables in the datasets, we also calculate the total length of the overloaded links.

In order to calculate the average additional investment of lv cables and the number of distribution transformers per household in this example neighborhood, we normalize the heat pump integration effects with the number of households N. For the distribution transformers, we see that it becomes overloaded when $h_i = 0.2$ thus its investment decision becomes a step function. Table presents the average investments per customer in the example grid. To assess the whole Netherlands, we can scale up this average neighborhood for each city and their ambition for the heat pump integration.

Table S1 Average investment length of lv cables and distribution transformers per household versus the probability of integration of heat pumps

Probability of integration of heat pumps h_i	Additional investment length of lv cables (m)	Additional investment of distribution transformers (units)
0	0	0
0.1	0.04	0
0.2	0.42	0.0011
0.3	0.42	0.0011
0.4	0.44	0.0011
0.5	0.45	0.0011
0.6	0.47	0.0011
0.7	0.48	0.0011
0.8	0.5	0.0011
0.9	0.51	0.0011
1	0.52	0.0011

A similar analysis has been done also for the hybrid heat pumps, with the assumption that the heat pump peak load is 3 kw (due to the switching to gas). Compared to the full electric heat pumps, we see the transformer is overloaded when $h_i = 0.3$ and the additional investments in the lv cables are slightly lower.

Table S2 Average investment length of lv cables and distribution transformers per household versus the probability of integration of hybrid heat pumps

Probability of integration of hybrid heat pumps h_i	Additional investment length of lv cables (m)	Additional investment of distribution transformers (units)
0	0	0
0.1	0.01	0
0.2	0.05	0
0.3	0.42	0.0011
0.4	0.42	0.0011
0.5	0.44	0.0011
0.6	0.45	0.0011
0.7	0.45	0.0011
0.8	0.47	0.0011
0.9	0.47	0.0011
1	0.49	0.0011

Supplementary Information II – Model input data

Table S3a, mean lifetimes for each low-carbon heating technology subcomponent (years):

	Building adjustments	Insulation materials	Infrastructure	Electricity and heat production	Sources
HT heating networks	25	75	50	20	[6,7]

LT heating networks	25	75	50	30	[6,8]
Heat pumps	25	75	40	25	[9–12]
Hybrid heat pumps	25	75	40	25	[9–12]

Table S3b, Weibull function parameters used in the Dynamic Stock Model based on the mean lifetimes from Table S3a:

	Building adjustments		Insulation materials		Infrastructure		Electricity and heat production	
	Scale	Shape	Scale	Shape	Scale	Shape	Scale	Shape
HT heating networks	2	28	67	2	2	57	2	23
LT heating networks	2	28	67	2	2	57	2	34
Heat pumps	2	28	67	2	2	45	2	28
Hybrid heat pumps	2	28	67	2	2	45	2	28

Table S4a, distribution of market share of low-carbon heating technologies for the Dutch built environment in 2050, based on the *warmtescenario* report by Berenschot [13]:

	Scenario 1 (Mix LT+ heat pump)	Scenario 2 (High heat pump)	Scenario 3 (High hybrid heat pump)
HT heating networks	5 %	5 %	10 %
LT heating networks	40 %	20 %	25 %
Heat pumps	35 %	55 %	25 %
Hybrid heat pumps	20 %	20 %	40 %

Table S4b, electricity generation composition for each scenario in 2050, based on the *klimaatneutrale energiescenarios* report by Berenschot [14], [15,16]:

	Scenario 1 (Mix LT + heat pump)	Scenario 2 (High heat pump)	Scenario 3 (High hybrid heat pump)
Biogas power plant	26 %	35 %	31 %
Wind onshore	10 %	7 %	12 %
Wind offshore	26 %	20 %	21 %
Solar power (PV)	38 %	38 %	36 %

Table S5a and S5b are input data used from from our previous paper for the calculation of the operational emissions over time [17].

Table S5a, CO_2 intensity per kWh of supplied heat for heating networks and heat pumps sources [18] [19]:

	Gram CO_2/GJ	gram CO_2/kWh heat	CO_2 intensity (natural gas = 1)	Tempera ture
Natural gas		192.8	1	N/A
Biomass	13000	46.8	0.24	LT
Waste heat without additional burning	8800	31.7	0.16	LT
Geothermal	25050	90.1	0.47	LT
Heat from burning waste	26000	93.6	0.49	HT
Waste heat Tata Steel	26000	93.6	0.49	HT
Waste heat from gas fired power plant	32000	115.2	0.60	HT
Waste heat from coal fired power plant	45000	162.0	0.84	HT

Table S5b, CO_2 intensity per kWh of supplied heat for heat pumps (COP = 3.5):

	Gram CO_2/kWh electricity	gram CO_2/kWh heat	CO_2 intensity (natural gas = 1)
PV	50	14.3	0.07

‘Grey’ electricity	365.83	104.5	0.54

Table S6, materials included and quantified in the model:

Other materials	Metals	Plastics
Cement	Aluminium	ABS
Ceramic brick	Brass	HDPE
Concrete	Bronze	PE
Limestone	Cast iron	Polyurethane (foam)
Sand	Copper	PVC
Wood fibreboard	Galvanized steel	Synthetic rubber
Mineral wool	Stainless steel	Glass fiber
	Steel	Polystyrene
	Nickel	
	Manganese	
	Chromium	
	Molybdenum	
	Tungsten	
	Niobium	
	Vanadium	
	Titanium	
	Cobalt	
	Tantalum	
	Neodymium	

Supplementary Information III – Model output data

DSM base scenario 1.2.xlsx

DSM base scenario 2.2.xlsx

DSM base scenario 3.2.xlsx

Materials_per_technology_v3.xlsx

References

1. IEEE. Resources – IEEE PES Test Feeder. https://cmte.ieee.org/pes-testfeeders/resources/ (2020) (accessed on 2021-03-02).

2. NEDU. Verbruiksprofielen. NEDU https://www.nedu.nl/documenten/verbruiksprofielen/ (2020) (accessed on 2021-03-02).

3. Cetinay, H., Kuipers, F. A. & Van Mieghem, P. A Topological Investigation of Power Flow. IEEE Syst. J. 12, 2524–2532 (2018).

4. Cetinay, H., Soltan, S., Kuipers, F. A., Zussman, G. & Van Mieghem, P. Analyzing Cascading Failures in Power Grids under the AC and DC Power Flow Models. ACM SIGMETRICS Perform. Eval. Rev. 45, 198–203 (2018).

5. Nyers, J. & Nyers, A. COP of heating-cooling system with heat pump. EXPRES 2011 - 3rd IEEE International Symposium on Exploitation of Renewable Energy Sources, Proceedings 21 (2011). doi:10.1109/EXPRES.2011.5741809.

6. Oliver-Solà, J., Gabarrell, X. & Rieradevall, J. Environmental impacts of the infrastructure for district heating in urban neighbourhoods. Energy Policy 37, 4711–4719 (2009).

7. Sullivan, J. Life-cycle analysis results of geothermal systems in comparison to other power systems. https://www.osti.gov/biblio/993694-wlvuul/ (2010).

8. Basosi, R. et al. Life Cycle Analysis of a Geothermal Power Plant: Comparison of the Environmental Performance with Other Renewable Energy Systems. Sustainability 12, 2786 (2020).

9. Greening, B. & Azapagic, A. Domestic heat pumps: Life cycle environmental impacts and potential implications for the UK. Energy 39, 205–217 (2012).

10. Jorge, R. S., Hawkins, T. R. & Hertwich, E. G. Life cycle assessment of electricity transmission and distribution—part 2: transformers and substation equipment. Int. J. Life Cycle Assess. 17, 184–191 (2012).

11. Spath, P. L. & Mann, M. K. Life Cycle Assessment of a Natural Gas Combined Cycle Power Generation System. NREL/TP-570-27715, 776930 http://www.osti.gov/servlets/purl/776930/ (2000) doi:10.2172/776930.

12. Vestas. Life cycle assessment of electricity production from an onshore V117-3.45 MW wind plant. 137 (2019).

13. Berenschot. Het warmtescenario. https://www.berenschot.nl/media/352kcpid/cases-het_warmtescenario.pdf (accessed on 2021-03-02).

14. Berenschot. klimaatneutrale energiescenario's 2050. https://www.berenschot.nl/media/hl4dygfq/rapport_klimaatneutrale_energiescenario_s_2050_2.pdf (accessed on 2021-03-02).

15. Rijksoverheid. Energierapport - transitie naar duurzaam. https://www.rijksoverheid.nl/documenten/rapporten/2016/01/18/energierapport-transitie-naar-duurzaam (accessed on 2021-03-02).

16. PBL. Achtergronddocument Effecten Ontwerp Klimaatakkoord: Elektriciteit. https://www.pbl.nl/publicaties/achtergronddocument-effecten-ontwerp-klimaatakkoord-elektriciteit (accessed on 2021-03-02).

17. Verhagen, T. J., Voet, E. van der & Sprecher, B. Alternatives for natural-gas-based heating systems: A quantitative GIS-based analysis of climate impacts and financial feasibility. J. Ind. Ecol. n/a, (2020).

18. MRA & TNO. De duurzaamheid van warmtenetten. http://warmteiscool.nl/wp-content/uploads/sites/17/2017/07/20170298-Brochure-A4-los1.pdf (accessed on 2021-03-02).

19. Stimular. CO2 Footprint - Milieubarometer website. https://www.milieubarometer.nl/CO2-footprints/co2-footprint/actuele-co2-parameters-2016/ (accessed on 2021-03-02).
